# Neonatal outcomes in relation to sex differences: a national cohort survey in Taiwan

**DOI:** 10.1186/s13293-015-0052-8

**Published:** 2015-12-09

**Authors:** Yi-Hao Weng, Chun-Yuh Yang, Ya-Wen Chiu

**Affiliations:** Division of Neonatology, Department of Pediatrics, Chang Gung Memorial Hospital, Chang Gung University College of Medicine, 199 Dunhua North Road, Taipei, 105 Taiwan; Department of Public Health, Kaohsiung Medical University, 100 Shih-Chuan 1st Road, Kaohsiung, 80708 Taiwan; Master Program in Global Health and Development, Health Policy and Care Research Center, College of Public Health and Nutrition, Taipei Medical University, 250 Wu-Hsing Street, Taipei, 110 Taiwan

**Keywords:** Sex ratio, Gender, Cesarean section, Birth weight, Gestational age, Birth outcome, Congenital anomaly

## Abstract

**Background:**

An extensive assessment investigating the association between sex differences and neonatal outcomes is lacking. In the current study, we estimated the correlation of gender with adverse birth outcomes in a large cohort population.

**Methods:**

National population-based data containing maternal and neonatal information in 2001 to 2010 were derived from the Health Promotion Administration, Taiwan. Singletons without high-risk pregnancy were further analyzed for the sex ratio of live births in relation to neonatal outcomes—including preterm birth, birth weight, neonatal death, delivery mode, and congenital anomaly. A multivariate logistic regression model was used to adjust for possible confounders.

**Results:**

In total, 2,123,100 births were valid for the analysis. Overall, the sex ratio at birth (male/female) was 1.096. Compared to multiple births, the sex ratio was significantly higher with singleton births (*p* < 0.001). Among multiple births, the incidence of stillbirths was significantly higher in males than in females (*p* < 0.05). The sex ratio at gestational age (GA) <37 weeks was 1.332, and it declined proportionally with a rise in the GA to 0.899 at GA of ≥41 weeks. In contrast, the sex ratio was 0.850 at birth weight <3000 g, and it rose proportionally with a rise in the birth weight to 1.902 at birth weight ≥4000 g (macrosomia). Operative delivery was more common in males than in females (*p* < 0.001). The regression analysis showed greater risks of preterm birth, macrosomia, operative delivery, neonatal death, and congenital anomaly among male newborns.

**Conclusions:**

Male gender carried higher risks of adverse neonatal outcomes, including preterm birth, macrosomia, operative delivery, neonatal death, and congenital anomaly. The data have clinical implications on health surveillance for plotting strategies in response to the unbalanced sex ratio in relation to the boy preference.

## Background

Males at birth outnumber females throughout the world [1]. The average sex ratio at birth, defined as the ratio of newly born male to female neonates in a population, is around 1.05 [2]. The sex ratio is associated with a variety of factors. The maternal age or birth order may alter the sex ratio at birth [2–5]. In addition, nutritional deprivation or exposure to certain chemicals during pregnancy may lead to a decline in the sex ratio at birth [6, 7]. The hypothesis of these alternations is that male fetuses are more vulnerable to environmental stimulation [8–12]. Socioeconomic parameters, such as a preference for boys, are also regarded as important reasons influencing the sex ratio [13]. With the advance of assisted reproductive technologies, sex-selective abortions have been blamed as a major factor causing gender inequity at birth [14, 15].

Although a number of studies investigated the association between sex differences and adverse pregnancy outcomes [16–18], little research has focused on correlations of the sex ratio at birth with neonatal conditions [4]. It is not clear whether a change in the sex ratio may have impacts on neonatal outcomes. In this retrospective cohort study, we explored nationwide population-based data of over 2 million births. Our results provide clinical implications of outcomes in relation to the sex at birth.

## Methods

### Data source

The targets of this population-based study were all births from 1 January 2001 to 31 December 2010 in Taiwan. Maternal and neonatal data were derived from the birth notification system, a database established by the Health Promotion Administration, Ministry of Health and Welfare, Taiwan [19]. Medical organizations and midwives have to report all births to this system via an online reporting system within 7 days. If there are changes in the reported data, revisions via the online reporting system are mandatory within 60 days. The Health Promotion Administration provided maternal and neonatal data and approved the use in this study. The study protocol was approved by the Research Ethics Committee of the National Health Research Institutes in Taiwan. All records of participants were anonymized and de-identified prior to analysis.

### Neonatal outcomes

Neonatal outcomes were assessed by the following seven parameters: multiple birth, stillbirth, neonatal death, congenital anomaly, gestational age (GA), delivery mode, and birth weight (low birth weight and macrosomia). Stillbirth was defined as the death of a fetus in the ≥20th week of gestation [20]. Congenital anomaly included any major abnormality of the central nervous, craniofacial, cardiovascular, digestive, urogenital, musculoskeletal, respiratory systems, or chromosome. Minor anomaly, such as a skin tag, was not recorded in this database. Preterm birth was defined as GA <37 weeks. Delivery mode was classified into cesarean section (CS) and vaginal delivery. Operative delivery was defined as delivery mode of either CS or vaginal delivery with the use of forceps or vacuum. Low birth weight was defined as a birth weight <2500 g. Macrosomia was defined as a birth weight ≥4000 g.

### Population for analyses

All births with an identified gender were included when estimating correlations of the sex ratio with stillbirths and multiple births. Otherwise, cases of stillbirths, high risk pregnancy, or multiple births were excluded when measuring correlations of the sex ratio with the following five outcomes: GA, birth weight, neonatal death, congenital anomaly, and delivery mode. High-risk pregnancy was defined as maternal conditions that threaten the health or life of the mother or her fetus, such as anemia (hematocrit <30 % or hemoglobin <10 g/dL), cardiac disease, pulmonary disease, diabetes, syphilis, polyhydramnios (amniotic fluid index (AFI) ≥24 cm or deepest vertical pool (DP) ≥8 cm) or oligohydramnios (AFI ≤5 cm or DP ≤1 cm), hemoglobinopathy, chronic hypertension, pregnancy-induced hypertension, toxemia, pre-eclampsia, cervical incompetence, Rh disease, renal disease, having a preemie or a baby with birth weight ≥ 4000 g or <2500 g.

### Statistical analyses

Statistical analyses were conducted using a commercially available program (SPSS 19.0 for Windows, SPSS, Chicago, IL, USA). We first applied a chi-squared test to determine whether sex ratio was associated with neonatal outcomes—including GA, birth weight (low birth weight and macrosomia), neonatal death, congenital anomaly, and delivery mode (CS vs. vaginal delivery) [4, 16–18, 21]. Further, we selected potential confounders a priori and included them into the analytic model. A multivariate logistic regression model was used to estimate the risk of neonatal birth outcomes in relation to the sex ratio after adjusting for possible confounders. Factors associated with sex ratio in the univariate analysis were used as confounders for the multivariate logistic regression analysis. Significance was defined as *p* < 0.05. The odds ratio (OR) and 95 % confidence intervals (CI) were expressed after adjusting for the control variables.

## Results

**Fig. 1:**
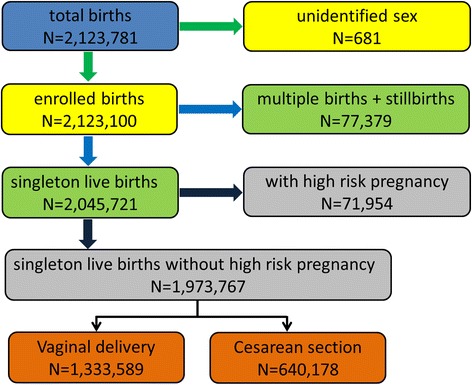
Assembly of study sample

Data on 2,123,781 births were collected in Taiwan from 2001 to 2010 (Fig. 1). We excluded 681 births with unidentified sex (ambiguous gender) and incomplete data, leaving 2,123,100 births for the analysis, including 1,109,989 males and 1,013,111 females (sex ratio at birth (male/female) = 1.096). Almost universal deliveries were accomplished by obstetricians in hospitals (68.14 %) and obstetric clinics (31.72 %). Only very few births were achieved by midwives (0.04 %), at home (0.08 %), or at other places (0.02 %).

### Multiple births

**Fig. 2:**
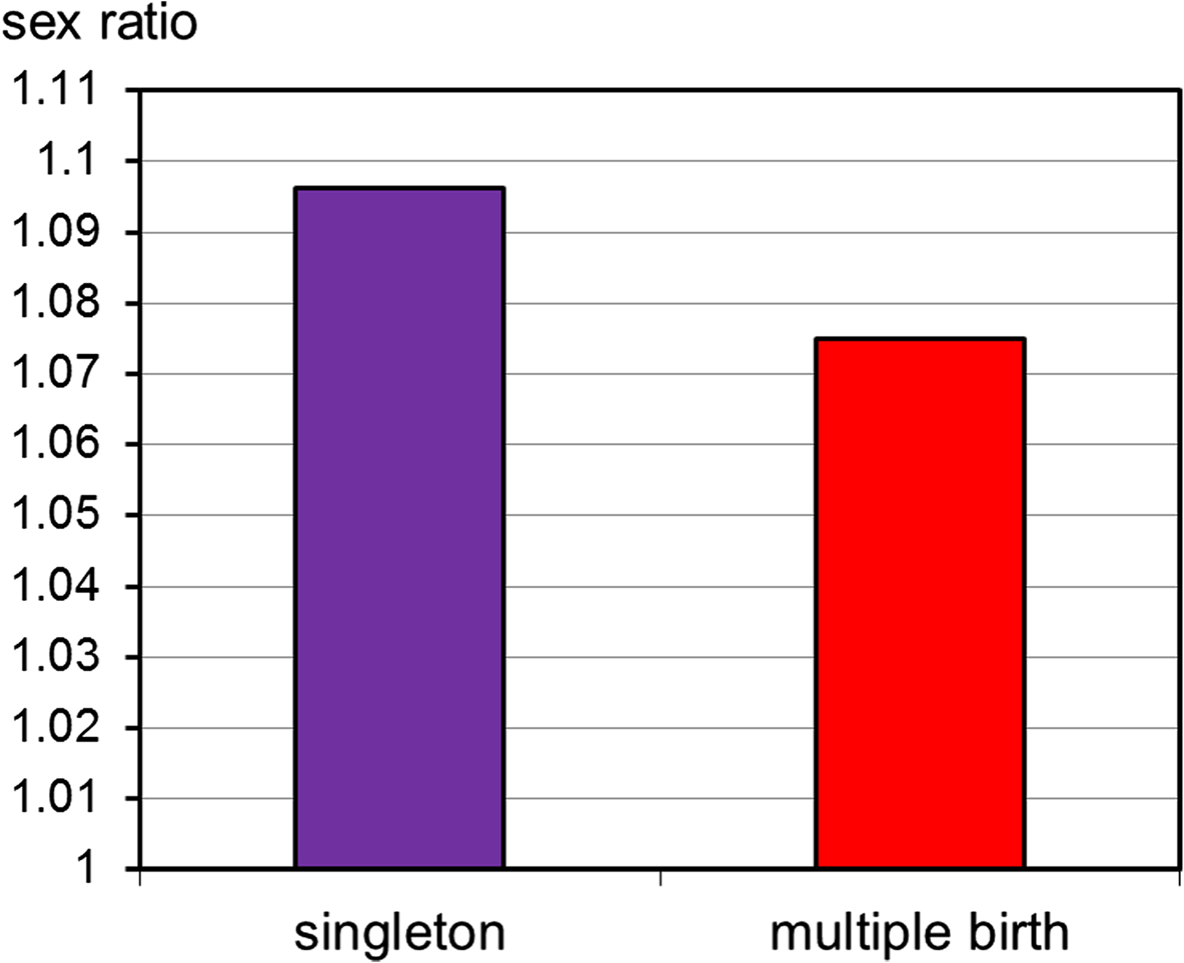
Sex ratios by single and multiple births among 2,123,100 births

The sex ratios were 1.096, 1.076, 1.065, and 0.733 for singletons, twins, triplets, and quadruplets, respectively. When compared to multiple births, the sex ratio was significantly higher for singleton births (*p* < 0.001) (Fig. 2).

### Stillbirths

**Fig. 3:**
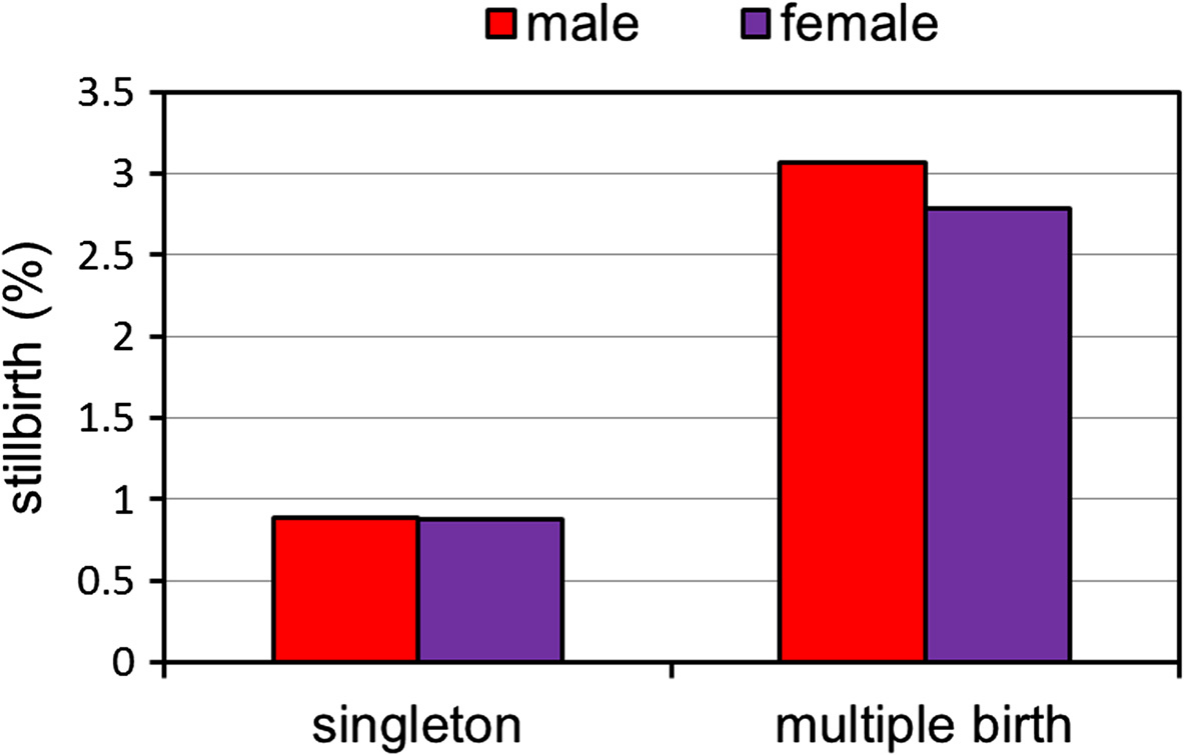
Rates of stillbirths by sex at birth among 2,123,100 births

The incidences of stillbirths were 0.88, 2.89, 4.34, and 11.54 % among singletons, twins, triplets, and quadruplets, respectively. Among multiple births, the incidence of stillbirths was significantly higher in males than females (*p* < 0.05) (Fig. 3). In contrast, there was no significant difference in the incidence of stillbirths between males and females among singletons.

### Singleton live births without high-risk pregnancy

In total, there were 2,045,721 singleton live births. We excluded 71,954 births with high-risk pregnancy, leaving a total of 1,973,767 births for further analysis (Fig. 1).

#### GA

The sex ratio among singleton live births without high-risk pregnancy was 1.096. There was a significant correlation of the sex ratio with GA (Table 1). The sex ratio was 1.356 for preterm birth, and it proportionally declined with an increase in the GA to the lowest level at GA ≥41 weeks.

**Table 1:** Gestational age (GA) in relation to sex ratio and neonatal outcomes among 1,973,767 singleton live births

GA (*w*)	Total			Neonatal death		Congenital anomaly		Low birth weight		Macrosomia	
	Male (*n*)	Female (*n*)	Sex ratio	%	Sex ratio	%	Sex ratio	%	Sex ratio	%	Sex ratio
≤24	804	666	1.207	77.48	1.320*	15.99	1.136	100	1.207	0.00	–
25	386	285	1.354	47.24	1.331	5.07	0.700*	100	1.354	0.00	–
26	455	316	1.440	31.00	1.276	4.67	1.118	100	1.440	0.00	–
27	540	438	1.233	22.29	1.396	3.48	0.789	100	1.233	0.00	–
28	683	534	1.279	14.71	1.594	3.12	0.652*	100	1.279	0.00	–
29	791	580	1.364	9.34	1.909	2.70	0.947	100	1.364	0.00	–
30	1145	881	1.300	5.92	1.400	2.86	1.148	99.70	1.298	0.00	–
31	1405	1032	1.361	4.10	1.273	2.95	1.483	98.89	1.358	0.00	–
32	2218	1581	1.403	2.21	1.270	2.47	1.410	95.97	1.408	0.00	–
33	3388	2371	1.429	1.96	1.457	1.86	1.432	88.82	1.384*	0.00	–
34	6298	4656	1.353	1.52	1.169	1.53	1.000*	72.08	1.222*	0.09	2.333
35	14,090	10,293	1.369	0.78	1.436	1.22	1.339	44.89	1.084*	0.32	1.026
36	42,246	31,262	1.351	0.35	1.421	0.79	1.750*	21.14	0.910*	0.64	1.597
37	139,442	109,431	1.274	0.17	1.434	0.56	1.455*	7.61	0.740*	0.99	1.876*
38	295,342	253,577	1.165	0.11	1.557*	0.45	1.521*	3.28	0.643*	1.45	2.173*
39	291,920	279,557	1.044	0.09	1.309*	0.43	1.394*	1.73	0.536*	2.18	2.053*
40	189,884	198,222	0.958	0.09	0.955	0.46	1.250*	0.96	0.521*	3.70	1.821*
41	38,268	42,722	0.896	0.13	0.946	0.59	1.154*	0.69	0.508*	5.65	1.669*
≥42	2933	3125	0.939	0.25	1.143	0.79	1.000	1.29	0.560*	8.40	1.495*
Total	1,032,238	941,529	1.096	0.27	1.341*	0.53	1.379*	5.31	0.863*	2.17	1.922*

*p < 0.05

#### Birth weight

**Fig. 4:**
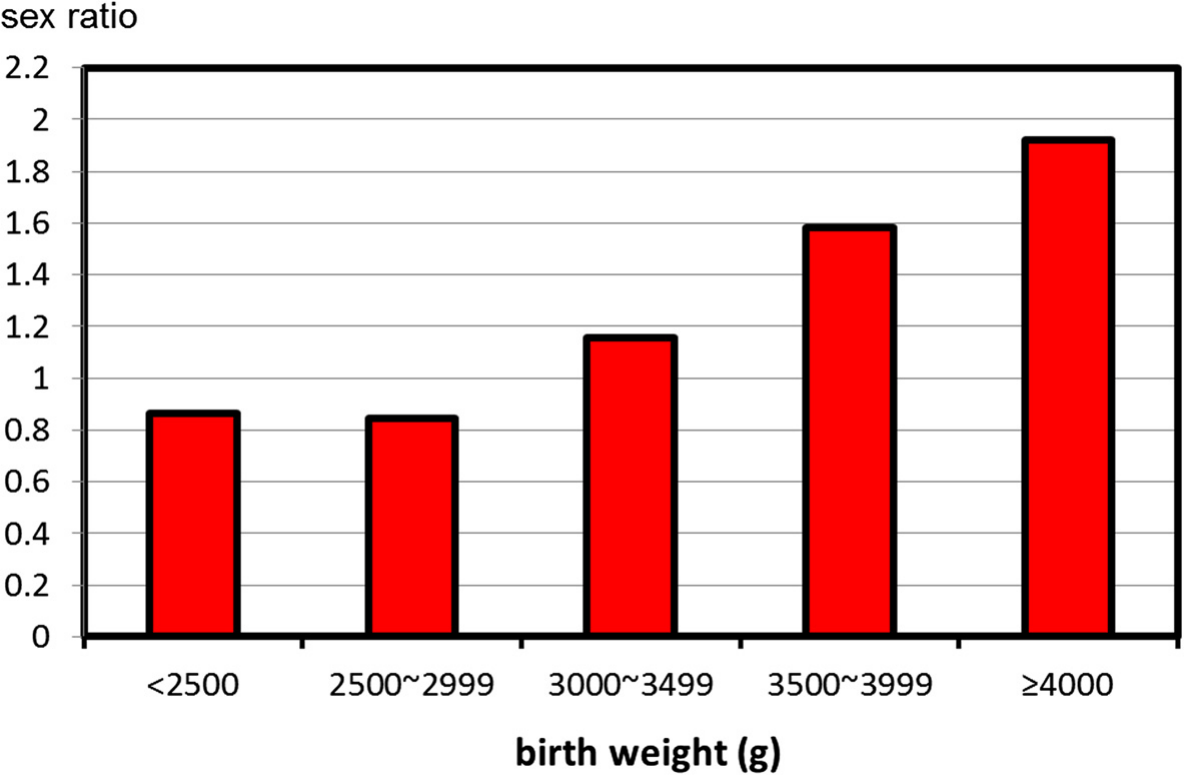
Sex ratios by birth weight among 1,973,767 singleton live births

The sex ratio by birth weight is demonstrated in Fig. 4. There was a significant correlation of the sex ratio with birth weight (*p* < 0.001). The sex ratio was 0.846 at birth weight <3000 g, and then it rose proportionally with an increase in the birth weight to 1.922 (the highest) at birth weight ≥4000 g.

Low birth weight infants were more common in females (5.98 %) than males (4.71 %) (*p* < 0.001). The incidence of low birth weight infants declined from GA of 29 weeks (100 %) to the lowest level at GA of 41 weeks (0.69 %), and then rose at GA of ≥42 weeks (1.29 %) (Table 1). In addition, there was a significant decrease in sex ratios of low birth weight infants among GA of ≥33 weeks when compared with those without low birth weight.

Macrosomia was more common in males (2.74 %) than females (1.56 %) (*p* < 0.001). The incidence of macrosomia increased proportionally from GA of 33 weeks (0 %) to the highest level at GA of ≥42 (8.40 %) (Table 1). Furthermore, there was a significant increase in sex ratios of macrosomia among GA of ≥37 weeks when compared with those without macrosomia.

#### Neonatal death

**Fig. 5:**
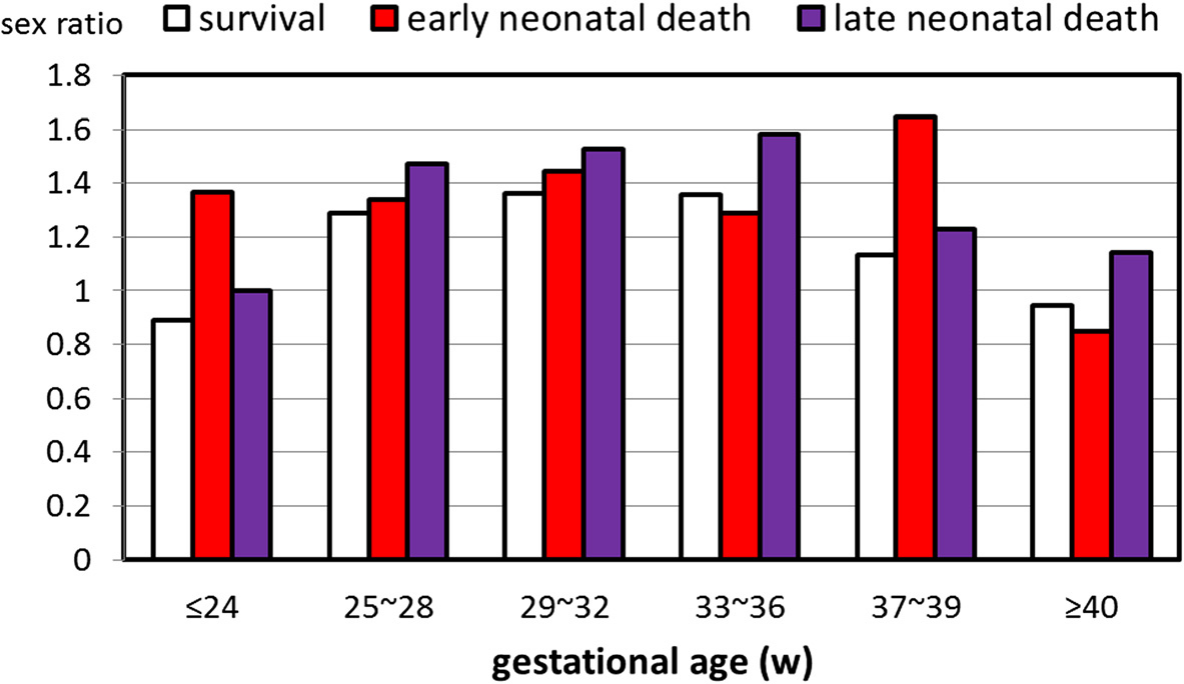
Sex ratios of survival and neonatal death among 1,973,767 singleton live births

Overall, 68.7 % of neonatal death occurred within 7 days of life (early neonatal death). There was an increasing risk of neonatal death among males compared to females, including early death and late death (*p* < 0.001). The sex ratio of neonatal death according to gestational age is illustrated in Fig. 5. When compared with neonatal survival, the sex ratios of early neonatal death were higher in GA of ≤24 and 37~39 weeks (*p* < 0.001).

Overall, the incidence of neonatal death was 0.27 % (Table 1). The incidence of neonatal death declined with an increase in the GA to the lowest level at GA of 40 weeks (0.09 %), and then increased to 0.25 % at GA ≥42 weeks. In addition, there was a significant increase in sex ratios of neonatal death among GA of ≤24, 38 and 39 weeks than those without neonatal death.

#### Congenital anomaly

Congenital anomaly was more common in males (0.58 %) than females (0.46 %) (*p* < 0.001). The incidence of congenital anomaly declined with an increase in the GA to the lowest level at GA of 39 weeks (0.43 %), and then gradually increased to 0.79 % at GA ≥42 weeks (Table 1). In addition, there was a significant increase in sex ratios of congenital anomaly among GA of 36~41 weeks than those without anomaly. In contrast, the sex ratios of congenital anomaly among GA of 25, 28, and 34 weeks significantly decreased than those without anomaly.

**Fig. 6:**
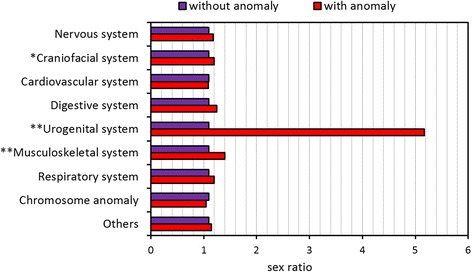
Sex ratios of congenital anomalies among 1,973,767 singleton live births. *p < 0.05. **p < 0.001

The sex ratios were significantly higher for neonates with abnormality in the craniofacial (*p* < 0.05), urogenital (*p* < 0.001), and musculoskeletal (*p* < 0.001) systems (Fig. 6). In particular, the sex ratio of neonates with abnormal urogenital system was 4.72 times that of neonates without urogenital anomaly. Furthermore, there was a trend of anomaly in the digestive system among males than females (*p* = 0.053).

#### Delivery mode

**Fig. 7:**
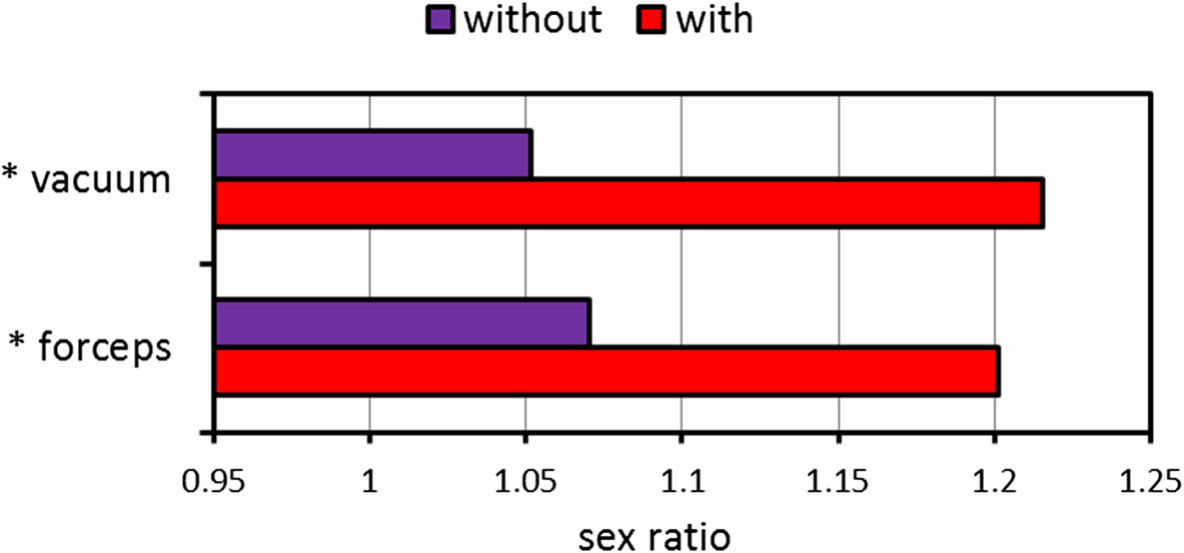
Sex ratios of operative delivery among 1,333,589 singleton live births by vaginal delivery. *p < 0.001

CS was more common in males (33.2 %) than females (31.6 %) (*p* < 0.001). In addition, the sex ratio of neonates with operative delivery (CS, forceps, and vacuum) was higher than that of neonates without operative delivery (*p* < 0.001). The sex ratio by operative vaginal delivery is further illustrated in Fig. 7. Among 1,333,589 vaginal deliveries, the sex ratio of vacuum deliveries was significantly higher than that of vaginal deliveries without the assistance of vacuum (*p* < 0.001). In addition, forceps were more often used in males than females (*p* < 0.001).

### Logistic regression analysis of birth outcome in relation to gender

The multivariate logistic regression model showed significant differences in the following six outcomes—including preterm birth, macrosomia, low birth weight, neonatal death, CS, and congenital anomaly. Preterm birth (OR = 1.498, 95 % CI = 1.478–1.517), macrosomia (OR = 1.749, 95 % CI = 1.714–1.785), neonatal death (OR = 1.231, 95 % CI = 1.182–1.280), CS (OR = 1.056, 95 % CI = 1.050–1.063), and congenital anomaly (OR = 1.280, 95 % CI = 1.230–1.331) were more common in males than females. In contrast, low birth weight infants were less common in males than females (OR = 0.645, 95 % CI = 0.636–0.654).

## Discussion

This study depicts that the sex differences are associated with neonatal outcomes, including GA, birth weight, neonatal death, congenital anomaly, and delivery mode. Our survey was based on a national population-based data registry between 2001 and 2010 to investigate the correlation of sex with neonatal outcomes. Our study eliminated high-risk pregnancy to avoid the possible bias from maternal factors. In addition, we used a logistic regression analysis to minimize the effects of confounders from neonatal factors. The data demonstrate that male gender is at greater risks for the following five neonatal outcomes: preterm birth, macrosomia, congenital anomaly, neonatal death, and operative delivery (CS, forceps delivery, or vacuum delivery).

First, our results indicate an increasing risk of preterm birth with male gender, which is similar with a couple of investigations [16, 17]. In our study, we further extend their inquiry by demonstrating GA-specific risks of sex differences. There is evidence showing that males are more vulnerable than females to suffer from ambient stressors during fetal development [4, 22]. Accordingly, we presume that preterm birth could be a subsequent outcome of males encountering fetal stress.

Second, macrosomia was more common in male neonates. In contrast, low birth weight was more common in female neonates. These findings are largely consistent with previous studies showing a higher risk of macrosomia and a low risk of small for GA among male infants [4, 17, 23]. It is not a surprising fact because growth is faster in male than in female fetuses.

Third, male neonates possess a greater risk of congenital anomaly. Our study further illustrated that a greatest risk on urogenital anomaly, followed by the musculoskeletal and craniofacial systems. The findings were largely in accordance with a number of studies reporting an elevated risk of congenital anomalies in males [24–27].

Fourth, operative deliveries were more common in male neonates, which is consistent with many studies [16, 18, 21, 28, 29]. It is probably because a large fetal weight, such as macrosomia, is an indication of CS for difficult labor [16, 29, 30]. Nevertheless, our logistic regression model showed a significantly higher rate of CS in males after controlling for cofactors, including birth weight. We speculated that sociopolitical behaviors also play an important role in the gender difference with CS [18, 28]. The preference for sons in Chinese parents may serve as the likely reason of CS [31], because they believe that the fate of their sons could be manipulated by the timing of birth via CS [32, 33].

Fifth, neonatal death was more common in males [34, 35]. In specific, early neonatal death contributed to a majority of neonatal death. Furthermore, males with GA ≤24 weeks carried higher risk of neonatal death, which is similar to a review showing that premature girls have better outcome than premature boys [36]. Delay of lung maturation among male fetuses may serve as an important factor to the sex difference in neonatal mortality [37]. In addition, our study demonstrated a greater risk of neonatal death among males at GA of 38 and 39 weeks. We suspected that, at least in part, congenital anomaly is associated with neonatal death of males [35].

Our study indicated that multiple births have a lower sex ratio than singletons, which was consistent with results from the previous studies [31, 38, 39]. In addition, we demonstrated a higher rate of stillbirths in males among multiple births. This finding was similar to those of previous studies which showed a greater risk for prenatal mortality among male fetuses [38, 40, 41]. The mechanism is probably due to multiple births being a significant stressor to male fetuses [22, 38], which will subsequently lead to stillbirth and a decline in the male birth number.

In our study, the sex ratio was higher than the average ratio in Western countries [2]. Selective abortion has been regarded as an important explanation for the skewed sex ratio in favor of males in some Asian countries, such as India and China [42–46]. However, an evidence-based study to prove this theory is lacking in Taiwan. In addition, the overall sex ratio at birth remained stable during the study period of 10 years, which is unlike a rising trend in the sex ratio in other Asian countries [31, 44, 47, 48]. The data suggest that, despite an unbalanced sex ratio at birth in Taiwan, the consequence of a preference for sons had only a slight effect.

There are limitations to this study. First, our study did not investigate the effects of assisted reproductive technologies. Nevertheless, there is no evidence to show any correlations of assisted reproductive technologies with the sex ratio in Taiwan [49, 50]. Second, we were unable to take parental socioeconomic factors, such as educational level, into account in our analyses because the information was not available in our database. Third, our study did not control for environmental factors in relation to the sex ratio [51]. We believe that the influence was small, because Taiwan is an island with no significant changes in outer exposures. Accordingly, people living in Taiwan might share a similar environment. Fourth, we are not certain that the delivery protocols were exactly the same. In Taiwan, the National Health Insurance Administration covers the expenses of all deliveries. It has established clinical guideline for delivery. Thus, we believe the majority of protocols employed in this study should be similar. Despite those limitations, there are two major strengths of this study. First, our study used a large nationwide sample size to analyze the sex ratio in relation to neonatal birth outcomes. The birth registration data used in this study were shown to have a good validity and reliability in birth outcomes [52, 53]. Second, our population-based source offered the potential to examine sex differences across the entire spectrum of neonatal birth outcomes.

## Conclusions

We identified male gender as an independent risk factor for adverse neonatal outcomes—including preterm birth, macrosomia, operative delivery, neonatal death, and congenital anomaly. The present study is consistent with the hypothesis that males are more vulnerable than females in the fetal environment. The data have clinical implications for health surveillance for plotting strategies in response to an unbalanced sex ratio. Parents with a preference for boys should take the greater risk of adverse neonatal outcomes with male gender into consideration.

## Abbreviations

CI: confidence intervals
CS: cesarean section
GA: gestational age
OR: odds ratio
